# Federated Learning for cross-jurisdictional analyses: A case study.

**DOI:** 10.23889/ijpds.v7i3.2026

**Published:** 2022-08-25

**Authors:** Mahmoud Azimaee, Lisa M. Lix

**Affiliations:** 1 Institute for Clinical Evaluation Sciences (ICES); 2 University of Manitoba

The objective of this project is to implement a harmonized artificial intelligence (AI)-based de-identification of free-text medical data across multiple Canadian jurisdictions. This federated learning approach will allow these jurisdictions to leverage each other’s data and resources while no individual-level data leaves the jurisdiction.

Federated Learning enables health data centers in different jurisdictions to collaborate in training machine learning models without sharing individual-level data. This approach will significantly reduce privacy and cybersecurity risks and barriers that are involved in sharing and moving data across different jurisdictions.

In a federated learning environment, machine learning models are trained on multiple data sources available in local data centers; local data are not shared to a central computing/analysis environment. Instead, parameters (such as model weights) are shared between these local data centers to generate a global model that will be shared and used by all participating data centers.

In this case study, four health research data centers in different Canadian provinces will take part in deployment of an AI-based application for de-identification of free-text data. The data centers are members of Health Data Research Network (HDRN) Canada. The deployment will include:

- harmonized annotation and labeling of local data,
- local training of entity recognition algorithms,
- integrating model weights from each data centers to create a global model
- development of license agreements between the participating data centers to allow sharing model weights

This is an ongoing project. The talk will demonstrate learning experiences, advantages, and challenges in a federated learning environment and explore the feasibility of transporting this approach to other multi-jurisdiction research networks.

